# Perceived social support and cognitive performance in preclinical autosomal dominant Alzheimer's disease

**DOI:** 10.1002/alz70857_107606

**Published:** 2025-12-24

**Authors:** Paula Ospina, Daniel Vasquez, Ana Y Baena, Susana Ramirez, Victoria Tirado, Eliana Henao, Victoria Zubiri, Claudia Aponte, Alejandro Guerrero, Jairo E. Martinez, Sonia Do Carmo, David Fernando Aguillón Niño, A. Claudio Cuello, Yakeel T. Quiroz

**Affiliations:** ^1^ Grupo de Neurociencias de Antioquia, Facultad de Medicina, Universidad de Antioquia, Medellín, Antioquia, Colombia; ^2^ Grupo de Neurociencias de Antioquia, Facultad de Medicina, Universidad de Antioquia, Medellín, Colombia; ^3^ Massachusetts General Hospital, Harvard Medical School, Boston, MA, USA; ^4^ Boston University, Boston, MA, USA; ^5^ McGill University, Montreal, QC, Canada

## Abstract

**Background:**

Perceived social support is an individual's sense of available emotional support from family and social networks. In neurodegenerative diseases, it has been explored as a potential protective factor against cognitive decline. Identifying modifiable factors is especially crucial for high‐risk populations. Individuals with autosomal dominant Alzheimer's disease (ADAD) typically develop early‐onset dementia, with research suggesting that certain factors may influence the timing of clinical onset. This study investigated the effects of perceived social support on cognitive performance in members of the Colombian kindred with the PSEN1 E280A mutation.

**Method:**

This study included 144 cognitively‐unimpaired individuals from the PSEN1 E280A cohort (64 carriers, 80 non‐carriers) from the Rita/NGF biomarker study. The mean age was 30.33(SD5.74) years for carriers and 34.38(SD9.23) for non‐carriers. Cognitive function was assessed using the MMSE and CERAD Word List Delayed Recall, while depression was measured with the Geriatric Depression Scale (GDS). Perceived social support was evaluated using the Multidimensional Scale of Perceived Social Support (MSPSS), which captures support from three sources: family, friends, and significant others. Group comparisons and associations were analyzed using the Mann‐Whitney‐Wilcoxon test and Spearman correlations.

**Results:**

Carriers had lower MMSE scores than non‐carriers (carriers: 26.3, SD 1.4; non‐carriers: 28.8, SD 1.3; *p* =  0.033) and reported more depressive symptoms (carriers: 2.8, SD 3.4; non‐carriers: 1.6, SD 2.2; *p* =  0.039). Additionally, carriers perceived lower overall social support (5.1 SD 1.3) compared to non‐carriers (5.6 SD 1.2; *p* =  0.014), with the difference largely driven by reduced support from family and significant others. These differences remained statistically significant even after controlling for depressive symptoms. However, perceived social support was not linked to cognition, age, or education.

**Conclusion:**

This study found significant differences in overall perceived social support between carriers and non‐carriers in individuals with autosomal dominant AD. Longitudinal follow‐up of this population is essential to further explore the relationship between perceived social support and other cognitive functions, such as executive function. Evaluating perceived support may help identify modifiable risk factors for dementia and inform interventions aimed at enhancing mental and brain health in individuals at high risk for AD.

